# Biocompatible Chitosan-Based Hydrogels for Bioabsorbable Wound Dressings

**DOI:** 10.3390/gels8020107

**Published:** 2022-02-10

**Authors:** Ramona Lungu, Maria-Alexandra Paun, Dragos Peptanariu, Daniela Ailincai, Luminita Marin, Mihai-Virgil Nichita, Vladimir-Alexandru Paun, Viorel-Puiu Paun

**Affiliations:** 1 “Petru Poni” Institute of Macromolecular Chemistry, Gr. Ghica Voda Alley, 41A, 700487 Iasi, Romania; lungu.ramona@icmpp.ro (R.L.); peptanariu.dragos@icmpp.ro (D.P.); ailincai.daniela@icmpp.ro (D.A.); lmarin@icmpp.ro (L.M.); 2 School of Engineering, Swiss Federal Institute of Technology (EPFL), 1015 Lausanne, Switzerland; maria-alexandra.paun@epfl.ch or; 3 Division Radio Monitoring and Equipment, Section Market Access and Conformity, Federal Office of Communications (OFCOM), 2501 Bienne, Switzerland; 4 Doctoral School, Faculty of Applied Sciences, University Politehnica of Bucharest, 060042 Bucharest, Romania; mihai_nichita9@yahoo.com; 5 Five Rescue Research Laboratory, 75004 Paris, France; vladimir.alexandru.paun@ieee.org; 6 Physics Department, Faculty of Applied Sciences, University Politehnica of Bucharest, 060042 Bucharest, Romania; 7 Academy of Romanian Scientists, 50085 Bucharest, Romania

**Keywords:** hydrogel, biocompatibility, antimicrobial activity, biodegradation, SEM image, fractal analysis

## Abstract

Supramolecular hydrogels based on chitosan and monoaldehydes are biomaterials with high potential for a multitude of bioapplications. This is due to the proper choice of the monoaldehyde that can tune the hydrogel properties for specific practices. In this conceptual framework, the present paper deals with the investigation of a hydrogel as bioabsorbable wound dressing. To this aim, chitosan was cross-linked with 2-formylphenylboronic acid to yield a hydrogel with antimicrobial activity. FTIR, NMR, and POM procedures have characterized the hydrogel from a structural and supramolecular point of view. At the same time, its biocompatibility and antimicrobial properties were also determined in vitro. Furthermore, in order to assess the bioabsorbable character, its biodegradation was investigated in vitro in the presence of lysosome in media of different pH, mimicking the wound exudate at different stages of healing. The biodegradation was monitored by gravimetrical measurements, SEM microscopy and fractal analyses of the images. The fractal dimension values and the lacunarity of SEM pictures were accurately calculated. All these successful investigations led to the conclusion that the tested materials are at the expected high standards.

## 1. Introduction

Wounds are a major health concern when they occur on large skin portions as a result of injury or illness, such as burns, chronic skin ulcers, venous stasis, or diabetes mellitus [1,2,3]. Long wound healing periods increase the risk of side effects such as infections, which lead to disfigurements and permanent physical disabilities, affecting the mental and socioeconomic status of patients [4]. Thus, many researchers have focused their attention to find solutions for rapid wound closure and developing an aesthetically satisfactory scar. To this aim, strategies including antibacterial ointments, synthetic growth factors, polyurethanes, polymeric hydrogels, and fiber dressings have been developed over the years [5,6,7,8]. Polymeric hydrogels showed the advantage of supporting a hydrated environment, adsorbing excess fluids [9,10,11]. Among them, those based on polysaccharides demonstrated non-toxic, biodegradable, and biocompatible properties, providing a good ability to improve the re-epithelization and acceleration of wound closure [12]. Chitosan biopolymer is a preferred polysaccharide to this aim, which demonstrated an acceleration of wound re-epithelialization due to its hemostasis potential and the stimulation of fibroblast proliferation, angiogenesis, regular collagen deposition, and the ability to favor the synthesis of natural hyaluronic acid (HA) at the wound site [13,14]. It was also reported that the healing ability is improved by the loading of antibacterial agents which prevent the bacterial invasion of wounds [15].

One major disadvantage of wound dressings is their adhesion to lesions, requiring mechanical debridement, which is damaging for the newly formed tissue and traumatic for the patient. To overcome this issue, the use of biodegradable chitosan for wound dressings is advantageous because it can be adsorbed into the skin during the re-epithelization process. Data in the literature show that during the healing period, the pH of wound exudate is a dynamic parameter, increasing from 8.5 to 10 in the first four days after the wound occurrence and decreasing slowly to 5.5 (the pH of the normal dermis) up to the total closure of wound [16]. However, even though a plethora of studies have been carried on chitosan-based materials, less attention has been directed to the influence of pH on their degradation.

In this light, the goal of this study was to investigate the biodegradation rate of a chitosan-based hydrogel as a function of the pH exudate over the wound healing period. To fulfill this objective, a chitosan-based hydrogel suitable for wound healing, exerting strong antimicrobial activity and excellent biocompatibility, was synthetized, and its enzymatic biodegradation rate as a pH function was monitored by gravimetrical measurements and SEM. A fractal theoretical application has been specially developed to support the quantitative investigation of SEM images and develop a better understanding of the biodegradation mechanism.

## 2. Experimental Investigation

### 2.1. Materials

Low-molecular-weight chitosan (178 kDa calculated by viscosimetry and a degree of deacetylation of 85% calculated from ^1^H-NMR [17]), 2-formyl-phenyl-boronic acid (**2FPBA**) (95%), glacial acetic acid, sodium hydroxide and lysozyme 40,000 U/mL were purchased from Sigma-Aldrich and used without further purification. Phosphate-buffered solution (PBS), pH 7.4, was prepared in our laboratory, and the pH was further varied using small amounts of sodium hydroxide or glacial acetic acid.

### 2.2. Synthesis of the Hydrogel

The studied hydrogel has been synthetized reacting the chitosan with 2-formyl-phenyl-boronic acid in homogeneous medium by an acid condensation reaction, as follows: 60 mg of chitosan was added to 2 mL of 0.7% acetic acid aqueous solution, and stirred for 30 min at room temperature, up to chitosan’s complete dissolution. Furthermore, the chitosan solution was heated up to 55 °C, and then a 1 mL solution of 2-formylphenyl boronic acid in water (2.3%, *w*/*v*) was slowly dropwise under vigorous magnetic stirring (1500 rot/min). In less than 10 min, the reaction mixture transformed into a soft material, which passed the inverted tube test, indicating the formation of hydrogel. The hydrogel was kept at 55 °C, without stirring, for 3 h, in order to facilitate the shifting of the imination equilibrium to the products.

### 2.3. Equipment and Methods

The *freeze-drying* was performed with a LABCONCO FreeZone Freeze Dry System, at −50 °C, 1.510 mbar, for 24 h, after previously freezing in liquid nitrogen.

*ATR-FTIR spectra* were registered with a Bruker Vertex 70 Ettlingen FTIR spectrometer (Billerica, MA, USA), on pieces of lyophilized hydrogel. The spectrum was recorded in the 600–4000 cm^−1^ spectral range, with 32 scans at 4 cm^−1^ resolution, and processed with OPUS 6.5 software and OriginProBit9.

*^1^H-NMR spectra* were recorded on a Bruker Avance DRX 400 MHz Spectrometer (Billerica, MA, USA), at room temperature. To this aim, the hydrogel was prepared directly into the NMR tube, replacing the bi-distilled water with deuterated water. The spectrum was recorded at different moments, starting with the initial moment (t0) when the hydrogelation occurred up, to 72 h. The chemical shifts are reported as δ values (ppm) relative to the residual peak of deuterated water.

Polarized optical microscopy images were acquired with a Zeiss Axio Imager M2 microscope (Zeiss, Wetzlar, Germany) on hydrogels and xerogels. The changes in the hydrogel morphology were monitored with a field-emission scanning electron microscope, SEM EDAX-Quanta 200 (Waltham, MA, USA), operating at an acceleration voltage of 20 keV.

Cytotoxicity of the hydrogel was assessed on normal human dermal fibroblasts (NHDF, PromoCell, Heidelberg, Germany) by MTS assay using the CellTiter 96^®^ AQueous One Solution Cell Proliferation Assay (Promega, Madison, WI, USA), according to the manufacturer’s instructions, and a direct contact procedure, according to ISO 10993-5:2009(E), for the biologic evaluation of medical devices [17]. First, the cells were grown in alpha-MEM (Lonza, Basel, Switzerland) supplemented with 10% fetal bovine serum (FBS, Gibco, Thermo Fisher Scientific, Waltham, MA, USA) and 1% penicillin–streptomycin–amphotericin B mixture (10 K/10 K/25 μg, Lonza, Basel, Switzerland) in a humidified atmosphere with 5% CO_2_ at 37 °C. Furthermore, the cells were seeded at a density of 0.5 × 10^5^ cells/mL into 96-well tissue-culture-treated plates in 100 μL culture medium/well and allowed to adhere for 24 h. Cells were then incubated for another 24 h with 100 μL culture medium and 10 (±0.01) mg of hydrogel sample was obtained by serial dilution to reach concentrations of 2FPBA from 0.284% to 0.004438%. Before incubating, the hydrogel samples were exposed to UV light (253.7 nm) for 30 min. Control cells were incubated only with culture medium. The next day, the medium in the wells containing the tested materials was replaced with 100 μL fresh medium and MTS reagent (20 µL) was added 3 h prior to absorbance readings at 490 nm on a microplate reader (EnSight, PerkinElmer, Rodgau, Germany). Experiments were performed in triplicate and the viability of the cells when in contact with hydrogel samples was expressed as a percentage of the control cells’ viability. Graphical data are expressed as the means ± standard error of the mean.

Antimicrobial tests were performed on pieces of hydrogels against three reference strains: *Escherichia coli* ATCC 25922, *Staphylococcus aureus* ATCC 6583 and *Candida albicans* ATCC 10231 [18]. Shortly, the hydrogel films were put into contact with the pathogen and their growth inhibition was measured using a caliper.

### 2.4. Enzymatic Biodegradation Tests

Pieces of hydrogels weighting from 147 mg to 258 mg (corresponding to 4–7 mg xerogel) were incubated in lysozyme solution (376 U/mL) in saline PBS of different pH values (5.5, 7.4, 8.5, 9 and 10) or lysozyme solution (4830 U/mL) in saline PBS of pH = 8.5, at a xerogel/media ratio of 1 mg/1 mL. In order to monitor the hydrolytic degradability, one sample was immersed in a PBS solution of pH 8.5, without adding lysozyme. At different moments relevant for wound healing—1 h and 1, 3, 7 and 14 days—the hydrogel pieces were taken from the media, washed with distilled water in order to remove the salts from PBS, lyophilized, and weighted with an analytical balance in order to establish the mass loss, applying the equation: $mass\;loss=\frac{mi-mf}{mf}\times 100$, where *m_i_* is the weight of the initial xerogel and *m_f_* is the weight of the lyophilized hydrogel at different moments of investigation. The experiments were performed in triplicate, and the results are expressed in terms of the mass loss ± S.D. obtained from the three independent measurements. All the samples resulted from the experiment were subjected to SEM in order to analyze the changes impacted by biodegradation.

## 3. Results and Discussion

### 3.1. Structural and Supramolecular Characterization

**Figure 1:**
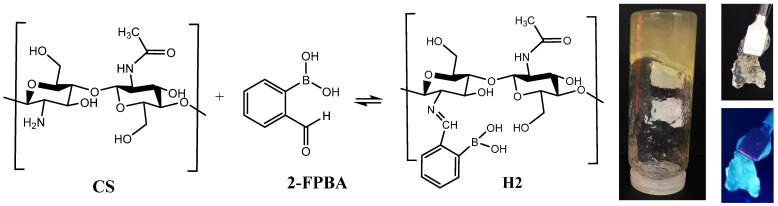
Imination reaction of chitosan with 2-formyl-phenyl-boronic acid and images of the obtained hydrogel, in normal light and when illuminated with a UV lamp.

An antimicrobial hydrogel was synthetized by the acid condensation reaction of chitosan with 2-formyl-phenyl-boronic acid in water (Figure 1), in view of investigating its biodegradability depending on pH, following the evolution of the pH exudate over the wound healing period. This hydrogel has been designed in view of applications for wound healing; thus, compared with the reported data [19], the synthetic procedure has been modified, i.e., the reaction was realized in a water biodispersant, avoiding the use of other organic solvents. The hydrogel state was confirmed by the inverted tube test (Figure 1).

**Figure 2:**
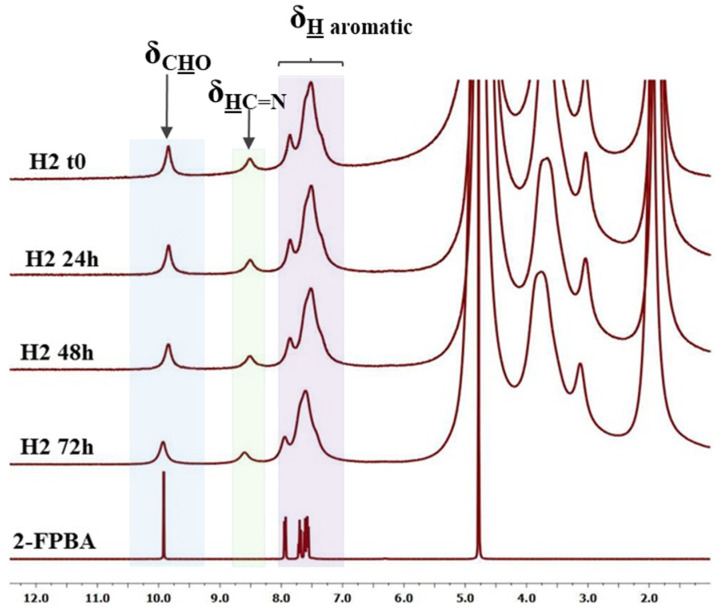
^1^H-NMR spectra of the hydrogel during hydrogelation, from hydrogel occurrence (t0) to 72 h, compared with the **2-FPBA** reference.

The structure and supramolecular architecture of the hydrogel were investigated by ^1^H-NMR, FTIR, and polarized light microscopy. As can be seen in Figure 2, ^1^H-NMR of the hydrogel showed both chemical shifts of imine and aldehyde protons, indicating that an imination equilibrium was established during the hydrogelation. Their ratio evolved over the hydrogelation period, according to an imination degree of 18.9% in the first few minutes, to 26.1% after one hour.

**Figure 3:**
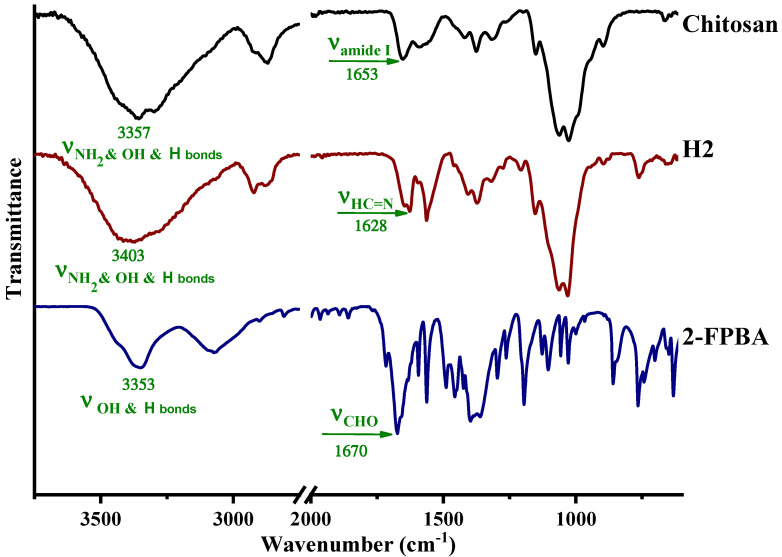
FTIR spectra of the lyophilized hydrogel compared with chitosan and **2-FPBA** references.

On the other hand, the FTIR spectrum on the corresponding lyophilized hydrogel showed the occurrence of the absorption band characteristic for the imine linkage at 1628 cm^−1^ [18,19,20,21,22] and no band specific for the aldehyde group [22,23,24], proving that the imination equilibrium was shifted to the products during the water removal process (Figure 3). This confirmed that the imination was a reversible process, which could be modified under the influence of external stimuli. In addition, the broad band characteristic for the vibration of hydroxyl and amine units in chitosan and the H-bonds prompted by them with the maxima at 3357 and 3300 cm^−1^, and that characteristic to hydroxyl units in aldehyde and the H-bonds prompted by them with the maximum at 3353 cm^−1^, shifted at higher wavenumbers in the hydrogel product (3403 cm^−1^), indicating the formation of a new H-bond environment [18,19]. Considering the structure of the chitosan and **2-FPBA** reagents and the imine product which resulted between them, it was estimated that the new H-bonds derived from the intermolecular interactions among the hydroxyl units of **2-FPBA** and the amine and hydroxyl units of chitosan on one hand, derived and from the intra- and intermolecular interactions between the hydroxyl groups and the nitrogen atom of the new imine units on the other hand. The stabilization of imine units by imino-boronate bonds [25] by “imine-clip” stabilization [26,27,28] is possible too.

**Figure 4:**
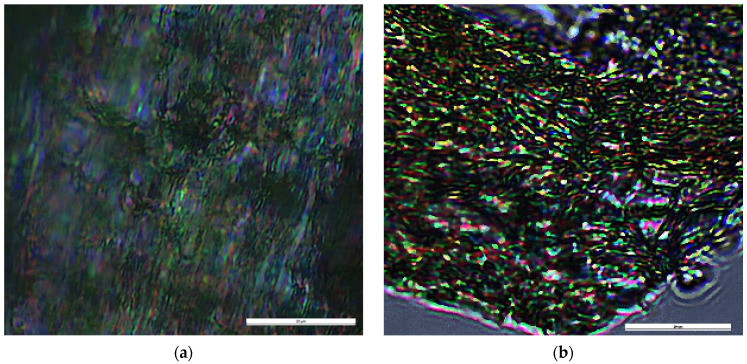
Images of the hydrogel under polarized light (scale bar: 20 μm), acquired on (**a**) thick and (**b**) thin sample of hydrogel.

POM images of the hydrogel showed bright birefringence with a banded texture identified for layered supramolecular architectures, proving that the new formed imine units self-ordered during the hydrogelation into a layered architecture pattern, similar to lyotropic liquid crystals (Figure 4) [29,30]. Furthermore, the hydrogel showed an emission of blue light when illuminated with a UV lamp (Figure 1), in line with the formation of supramolecular fluorophores [26]. This is in agreement with our previous studies, which proved that the hydrogelation of chitosan with monoaldehydes is possible due to an imination reaction followed by the self-assembly of the newly formed imine units into layered clusters playing the role of crosslinking nodes [26,30].

### 3.2. In Vitro Biologic Properties

**Figure 5:**
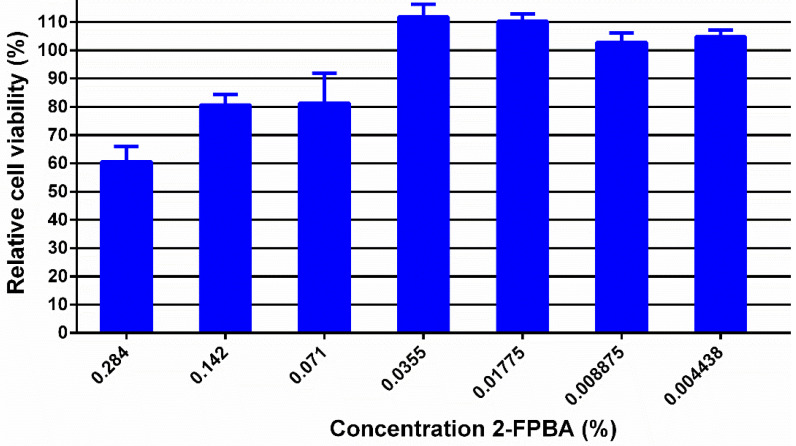
Cytotoxicity tests of hydrogels on normal human dermal fibroblasts.

This hydrogel was designed for wound healing; therefore, its cytotoxicity against normal human dermal fibroblasts (NHDF) was investigated in order to establish the **2-FPBA** level for which the hydrogels can be safely used in contact with tissues (Figure 5). To do this, the hydrogel was diluted to rich concentrations of **2-FPBA** in hydrogel from 0.284 up to 0.004438%. As can be seen, except for the concentration of 0.284%, the hydrogels showed cell viability higher than 70% which, according to ISO 10993-5:2009(E) for the biologic evaluation of medical devices, indicates that they can be safely used in bioapplications [17].

It should be stressed that the hydrogel showed strong antimicrobial activity against relevant pathogens, reaching inhibition zones of 9 mm (*S. aureus*), 15 mm (*E. coli*) and 17 mm (*C. albicans*) [18]. Moreover, for the 2FPBA concentration of 0.142% in hydrogel, the antifungal effect recorded a microbial burden reduction of 99.999% against the Candida species in 24 h, proving that, for this concentration, the hydrogels can successfully be applied on wounds to assure complete protection against infections [19]. The antimicrobial activity has been correlated with the reversibility of the imination, which favored the equilibrium shifting to the reagents once they were consumed in the pathogen killing process [18,19].

### 3.3. Biodegradation Investigation

**Figure 6:**
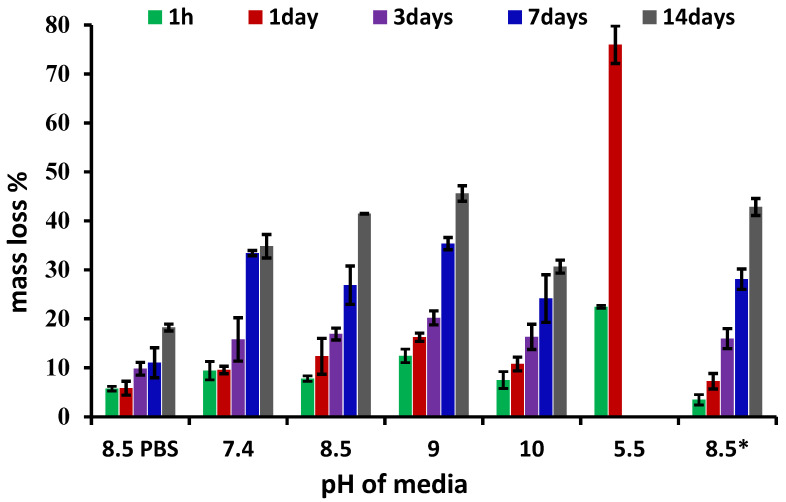
The mass loss of hydrogel over 14 days, in lysozyme media (376 U/mL) of different pH (* indicates a lysozyme concentration of 4830 U/mL; 8.5PBS indicates the absence of lysozyme).

In this line of thought, the rational question arising is to what extent the hydrogels can be applied on wounds without the need for traumatic debridement, while they are an active barrier against microbial infections. In this view, a biodegradation experiment was performed in media of different pH values, corresponding to that of pH exudate over the wound healing period. The results are presented in Figure 6. First, the clear influence of lysozyme on biodegradation can be seen, a mass loss up to 45% being reached compared with 17% in its absence. Furthermore, it is observed that the pH of the lysozyme medium clearly influenced the biodegradation rate. Thus, whereas in the medium of pH = 7.4 characteristic to the physiological environment the mass loss was 32%, in that of pH = 8.5 and 9, characteristic to the exudate of wounds in the first 4 days of healing, the mass loss increased to 42% and 45%, respectively. This is particularly important because this pH is favorable for the proliferation of infection [31] and faster biodegradation of the hydrogel indicates the faster release of the antimicrobial aldehyde assuring a self-defense environment. Interestingly, at pH 10, (characteristic for day 4 of the healing period), the biodegradation rate slowed down, reaching a mass loss of 30%. The increase in lysosome concentration, representative of infected tissues, inflicted a slightly increase in degradation, leading to a mass loss 43%. On the other hand, at pH = 5.5, which is characteristic for the normal dermis [32], the biodegradation abruptly amplified, leading to a mass loss of 75% in the first day, and totally vanishing by day 2. This suggests that the hydrogel will be rapidly adsorbed into the newly formed tissue, with no need for a traumatic debridement, favoring smooth tissue regeneration without trauma. This suggests wound healing without scars.

**Figure 7:**
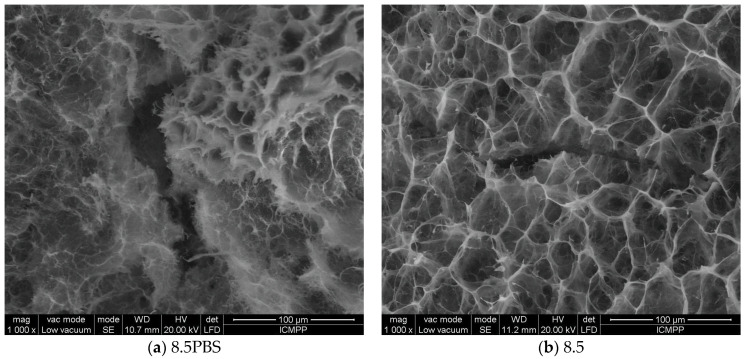

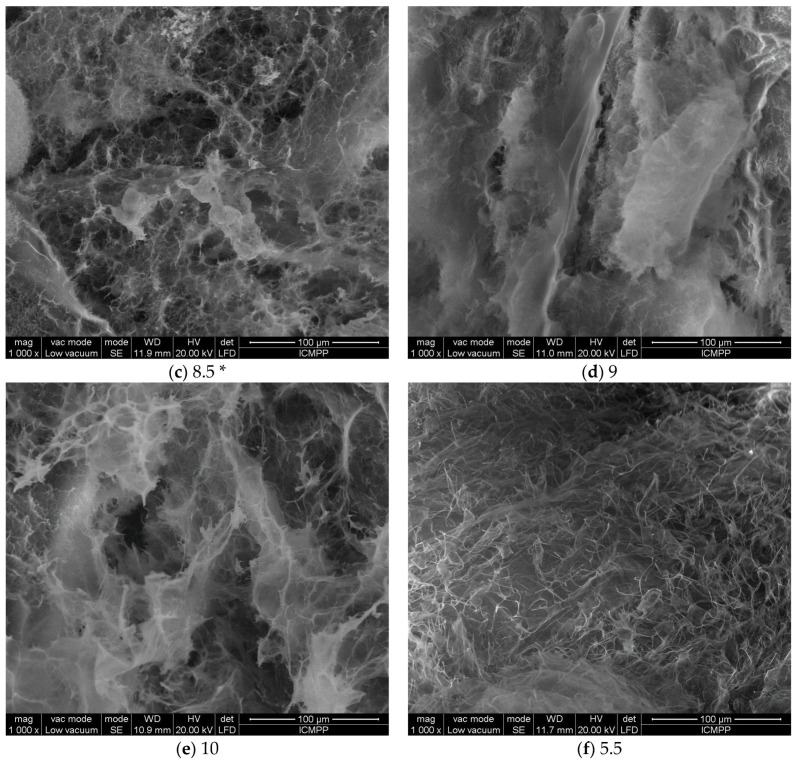
The morphology of the hydrogel after the enzymatic biodegradation over 14 days at different pH (**a**–**e**) and after the first day of biodegradation in medium of pH = 5.5 (**f**). (* indicates a lysozyme concentration of 4830 U/mL; 8.5PBS indicates the absence of lysozyme).

The evolution of the hydrogel morphology during the degradation was investigated by SEM (Figure 7).

In PBS, without enzyme, the pores of hydrogel appeared collapsed, and ruptures occurred, in line with the release of the aldehyde and partial dissolution of the chitosan. Interestingly, no such effect was observed at pH = 8.5 in the presence of a lysozyme concentration characteristic to the non-infected wounds in the first minutes after wound occurrence; the morphology appeared less affected. On the other hand, at pH = 9.5 and 10, characteristic to a wound exudate over the healing period of the first four days, which is determinant for the evolution of wound to a complete recovery of tissue, the hydrogel showed a loose morphology, in agreement with the massive mass loss. Furthermore, at pH = 5.5, characteristic for the normal dermis, massive biodegradation was evident, with the hydrogel morphology transforming into a fibrous one, suitable for tissue regeneration.

### 3.4. Application Results of Fractal and Lacunar Analysis Algorithms

Fractal geometry is the mathematical completion that Euclidean geometry and crystalline (or quasi-crystalline) symmetry were lacking. Fractal analysis, which is the main vein/lode exploited with this new way of thinking, works with two basic notions, namely, fractal size and lacunarity.

The fractal dimension alone does not characterize the object studied from a fractal point of view. There are many sets (mathematical objects) that have the same fractal dimension but a completely different spatial structure. Thus, two sets of Cantor type have the same fractal dimension, but differ in their lacunarity, another characteristic which is required, not only intuited, but also clearly defined.

Fractal analysis uses this term, respective lacunarity, to describe the uniformity of texture in images. It can also characterize the size of a space, the homogeneity of objects, and the rotational and translational invariance of an image.

A low gap coefficient refers to a homogeneity, defined as low (or reduced) lacunarity, which implies gaps of similar size and low rotation variance. On the other hand, a high coefficient of gaps refers to heterogeneity, defined as a large (or high) lacunarity, which implies a larger number of gaps.

**Figure 8:**
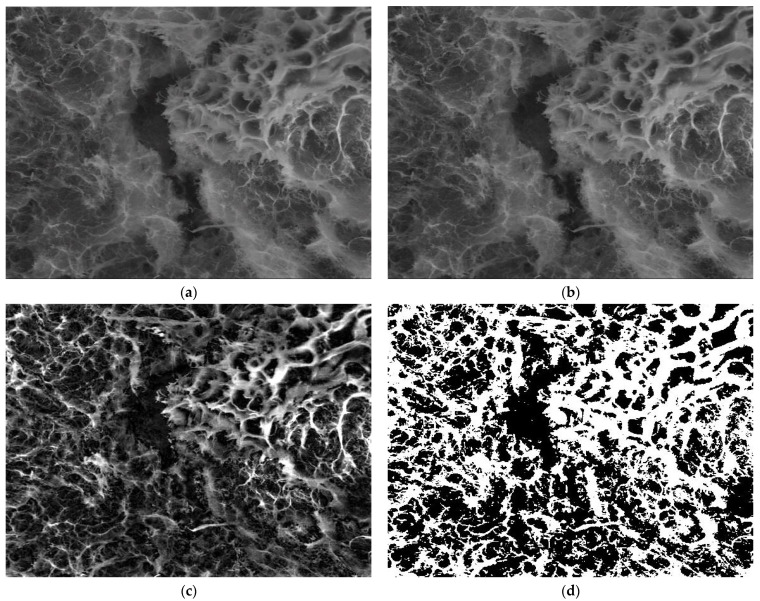

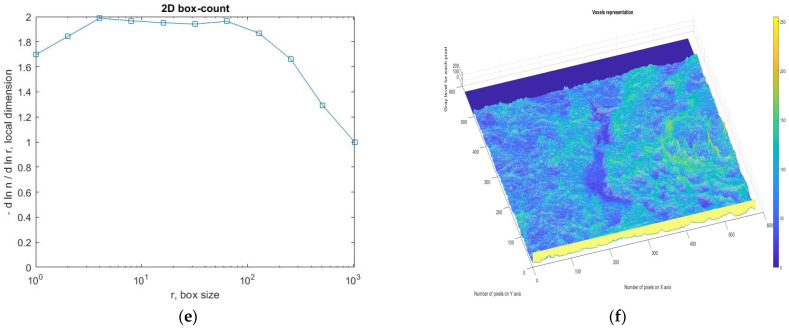
Fractal investigation of image A: (**a**) Black and white version of image A, (**b**) Grayscale version extracted from the background, (**c**) Grayscale version with luminance, (**d**) Binary version, (**e**) Box-count representation, (**f**) Voxel representation.

**Figure 9:**
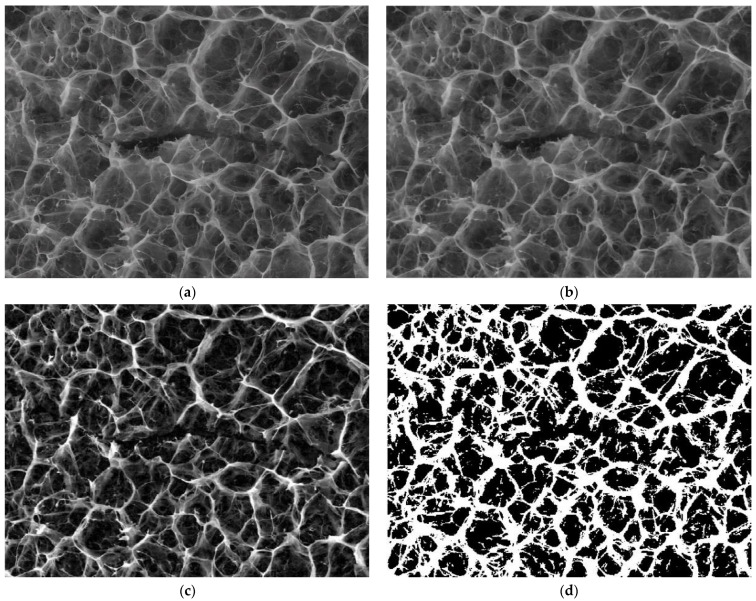

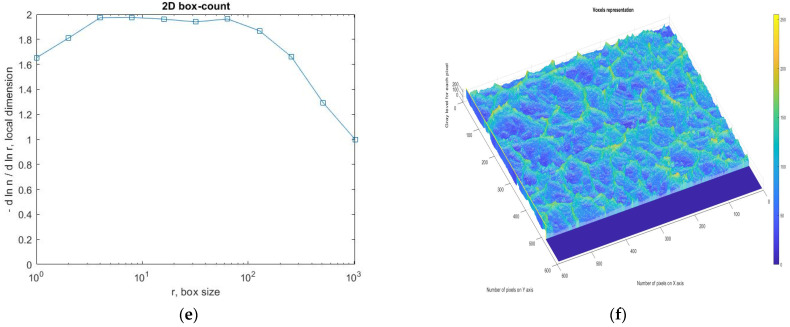
Fractal investigation of image B: (**a**) Black and white version of image B, (**b**) Grayscale version extracted from the background, (**c**) Grayscale version with luminance, (**d**) Binary version, (**e**) Box-count representation, (**f**) Voxel representation.

**Figure 10:**
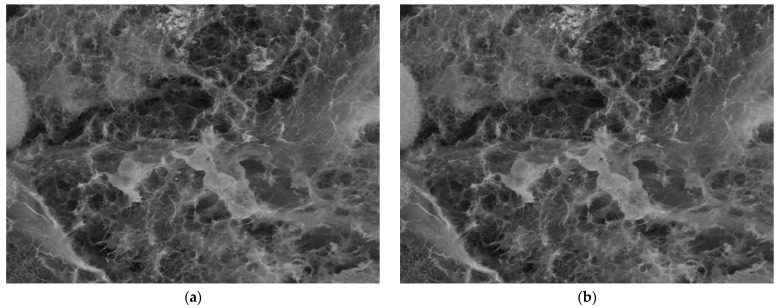

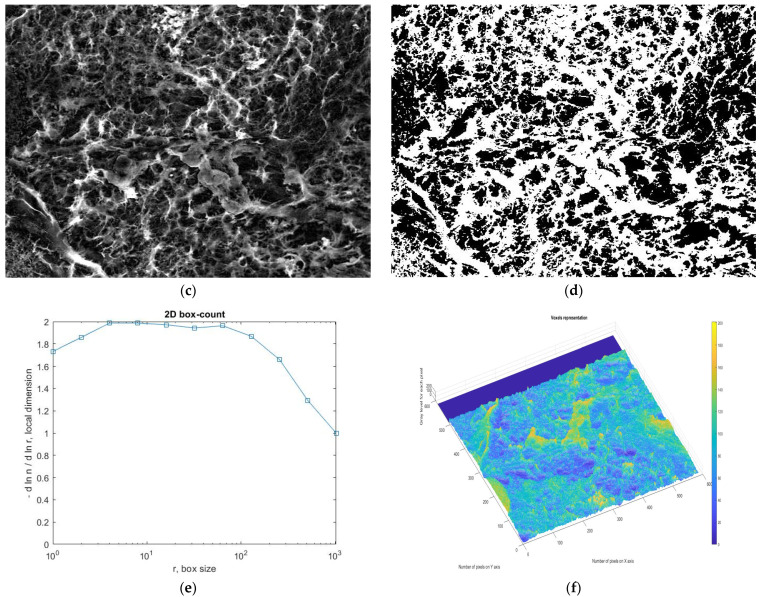
Fractal investigation of image C: (**a**) Black and white version of image C, (**b**) Grayscale version extracted from the background, (**c**) Grayscale version with luminance, (**d**) Binary version, (**e**) Box-count representation, (**f**) Voxel representation.

The SEM images of Figure 7, indicated by the abbreviation 8.5PBS (image A), 8.5 (image B) and 8.5 × (image C) were investigated by fractal analysis [33,34,35,36] in Figure 8, Figure 9 and Figure 10. The abbreviation 8.5PBS indicates the absence of lysozyme. In our images, we used a magnitude of 1000×.

The pictures were preprocessed in order to optimize the binary process (the luminance was removed from the original image). Binarization involved setting a threshold according to which the pixels (gray levels in the image) were set to 0 or 1 (55 for the first image, 60 for the second, and 57 for the third).

For image A, we determined the following values obtained by applying the fractal analysis procedure: the fractal dimension d = 1.9131 with a standard deviation of ±0.10211 and lacunarity λ = 0.5995.

For image B, we determined the following values obtained by applying the fractal analysis procedure: the fractal dimension d = 1.9171 with a standard deviation of ±0.10422 and lacunarity λ = 0.6581.

For image C, we determined the following values obtained by applying the fractal analysis procedure: the fractal dimension d = 1.9213 with a standard deviation of ±0.10675 and lacunarity λ = 0.6069.

## 4. Conclusions

A chitosan-based hydrogel with biocompatibility and antimicrobial properties suitable for wound healing has been synthetized by an acid condensation reaction with 2-forml-phenyl-boronic acid to yield imine units and their supramolecular ordering into ordered clusters with the role of crosslinking nodes.

The hydrogel proved to have a lack of toxicity against normal dermal human fibroblasts and antimicrobial activity against relevant pathogens, such as *E. coli*, *S. aureus* and *C. albicans*. The investigation of the hydrogel biodegradation as a function of the pH of the wound exudate over the healing period demonstrated that the biodegradation rate is modulated by pH over the healing period, and is favorable to re-epithelization and avoiding traumatic debridement.

The topographic assessments of SEM images of the hydrogels degraded in lysozyme media of different pH, based on the evaluation by fractal analysis, showed that the evolution of the values of fractal dimension and lacunarity (d = 1.9131 ± 0.10211 and lacunarity λ = 0.5995 for image A, fractal dimension d = 1.9171 ± 0.10422 and lacunarity λ = 0.6581 for image B and fractal dimension d = 1.9213 ± 0.10675 and lacunarity λ = 0.6069 for image C) fitted well on the values of mass loss, confirming once more that the biodegradation rate is modulated by pH.

## Data Availability

The data used to support the findings of this study cannot be accessed due to commercial confidentiality.
